# Observations on the Results of Removal of Diseased Uterine Appendages1Read before the Obstetrical and Gynecological Section of the Buffalo Academy of Medicine.

**Published:** 1894-02

**Authors:** M. A. Crockett

**Affiliations:** Assistant Gynecologist, Buffalo General Hospital; Instructor in Medical Department, University of Buffalo


### Buffalo Medical and Surgical Journal

Vol. XXXIII. FEBRUARY, 1894. No. 7.

### Original Communications.

### OBSERVATIONS ON THE RESULTS OF REMOVAL OF DISEASED UTERINE APPENDAGES.[1]

M. A. CROCKETT, A. B., M. D.

Assistant Gynecologist, Buffalo General Hospital ; Instructor in Medical Department, University of Buffalo.

THE appended table includes all the abdominal sections performed by me up to November 1, 1893.

Examination of this list shows that twenty of these operations were performed for inflammatory conditions of the uterine appendages, and it is from the clinical study of the results in these cases that I wish to draw a few conclusions and submit them to your consideration. If we adopt Pozzi's classification, we may divide these cases into non-cystic oöphoro-salpingitis (9) and cystic oöphoro-salpingitis (11). The latter division includes hydro-, hemato- and pyosalpinx, abscess of the ovary, etc. The first division comprises only the cases of chronic parenchymatous salpingitis and hypertrophic or sclerous oöphoritis.

1. Looking at this list of cases, it will be noticed, in the first place, that the removal of the uterine appendages, even in a case in which there is no pus, is not an absolutely safe operation. The fatal case of this series was one of the simplest operations, and, although every antiseptic precaution was taken, infection occurred. Where the weak link in the chain of antisepsis existed will never be known ; but the fact that such a link can exist in spite of the utmost care, should be borne in mind and should be duly considered in the advice given to the patient. Fortunately, such accidents are very rare.

2. As far as can be ascertained, none of the survivors were made worse for the operation. Every case was distinctly benefited,

1. Read before the Obstetrical and Gynecological Section of the Buffalo Academy of Medicine.

although very few of them can be put down as completely cured; that is, as being left without an ache or a pain.

This brings us to the subject as to what constitutes a cure following a surgical procedure. It should be considered carefully that most of these patients had been ill a long time, and that the operation was performed with the idea of relieving certain distinct symptoms. If those symptoms *were* relieved, then we may claim that the operation was justified.

One of the great faults committed by those condemning the removal of diseased tubes and ovaries, is that of assuming that these patients must necessarily be completely rejuvenated. No other surgical procedure is subjected to such a test.

In almost every chronic case, or in every case in which the operation is a severe one, there are certain influences remaining which retard or hinder the complete recovery. Let us consider some of the principal disadvantages under which these operations are performed, and which may be inseparable from the operation itself.

1. Ventral Hernia.

Every abdominal section which necessitates the use of a drainage-tube, must leave a weak spot in the abdominal wall. I know of two hernias following my operations, aside from the one which I sewed up. In surgical procedures of great urgency, this consideration is of little importance. But, in those cases in which operation is not imperative, this danger should be given full weight. To be sure, the closure of a small ventral opening is neither difficult nor dangerous, but patients who have once been through the discomforts of abdominal section, are very loath to submit themselves again to the surgeon's knife.

2. The impossibility of leaving the pelvis in an absolutely normal condition.

In cases in which large pus tubes or densely adherent appendages are removed, a more or less extensive raw surface is left behind. The intestines themselves may be injured over considerable areas of their peritoneal surface. Adhesions are thus produced which drag upon or compress the surrounding viscera, and interfere, to a greater or less degree, with the physiological functions of these organs. It is surprising that more trouble does not result from this cause when we see the extensive raw surfaces often left in the pelvis after completing some of these operations. There are cases reported where the abdomen has been opened a second time, in order to break up adhesions about a stump. If we cannot have a normal pelvis, we may at least substitute a lesser for a greater evil.

3. Neglect of treating all the disease.

Salpingitis is almost invariably an extension of endometritis. The roots of the trouble remain behind in the uterus. As may be seen, several of my cases came back complaining of symptoms of endometritis. It should be made an invariable rule to precede salpingo-oöphorectomy with curettement of the uterus, even if endometrial symptoms are not prominent. Some authorities advocate removal of the uterus in toto. Where a thoroughly diseased organ is present, such a step seems reasonable. Why should we expect very brilliant results when merely a portion of the diseased structures has been removed? The presence of a diseased uterus will account for many of the disappointments following salpingo-oöphorectomy.

Under this same head should be mentioned the neglect to fasten a displaced uterus in proper position.

4. The ultimate results of this operation are influenced very largely by the effect of long-standing tubal disease upon other organs, particularly the nervous system.

In those cases which have been accompanied by pain, due to irritation of the nerves radiating from the pelvis, it is reasonable to suppose that a certain amount of neuritis is produced. Even if we remove the irritant, we have left the more or less permanent changes in the nerves. It is well known that headache is produced by refractive errors in the eye, and it is often observed that after those errors are corrected by proper glasses, the headaches may continue for several months. Why should not the same thing occur after operation in cases of long-standing pelvic disease? Again, pain is supposed to be due to wave-like impressions along the nerves. After removal of the diseased appendages, the wind is lulled, but the waves continue, for a time at least. Many physicians seem to expect that a woman can suffer for years from pelvic disease, and that if, directly after the operation, she does not walk out of the hospital a well woman, the treatment has been a failure. Such an idea, to my mind, is absurd, and simply shows the lack of common sense manifested in discussing this subject.

5. Affection of other organs dependent or independent of disease in the pelvis.

This consideration is closely allied with the preceding. If, for years, nutrition and excretion have been faulty, it stands to reason that it will take time to bring our patient's health up to par. The surgeon can very properly take the ground that the removal of organs which are acting as a focus of irritation will allow a better opportunity for the vital processes of the body to pursue a natural course. These processes may have been permanently impaired, but it is from disease and not from operation.

6. Effect of long suffering and treatment on the patient's morale.

Women who have for years spent a portion of every day in considering what aches or pains they have, and whether this or that treatment does or does not relieve them, are very apt to develop a habit of excessive introspection. Then, if a surgeon makes the mistake of promising a miracle by an operation, he will merit his fate. After operation in such cases, every minute sensation is magnified into a pain, multiplied by the amount of disappointment experienced. I believe that a great many of those cases reported worse after operation are cases of this kind. I believe that the patient forgets how bad her condition was before, and that a careful, minute interrogation will show that although not cured, she is distinctly better. In other cases, the patient's statements are absolutely untrue. Because they are not entirely well, they will tell you that they are no better. We all see such people outside of surgery, and their statements should not be accepted without question.

7. Patient's subsequent occupation and condition of life.

One of my patients complains of backache. I find her occupation to be that of a cook in a restaurant, hours from 7 A. M. to 7 P. M., and work hard. I also find that this backache comes on about 4 P. M. I think that many of us, under the same circumstances, would have backache much earlier in the day. And yet, how easy for the non-operative enthusiast to hold this patient up as a failure, merely by omitting to state that previous to the operation she was unable to be about her own house.

What shall we say of the patients pursuing their vocations in the infected districts ? If such patients, six months after operation, are not entirely well, does it prove anything against the operation ?

Almost all hospital patients go back to a life of hard work ; work which often breaks down a healthy woman. Under these circumstances, I can easily imagine a patient not so well at the end of two years as at the end of one year, and here again is a splendid opportunity for those who wish to gather evidence against ophoro-salpingectomy. As will be seen later, it is not my intention to make any extravagant claims in favor of operation, but I do wish to expose some of the fallacies of the arguments of the other side. It seems to me that the mere fact that many of these patients whom I have reported are able at all to perform their present occupations, is evidence speaking well for the operative treatment.

8. Lack of skill and judgment on the part of the operator.

A few months ago I saw a patient whose abdomen had been opened and closed again, because there were adhesions between the ovary and rectum. This patient is not enthusiastic over surgical treatment, and the case is probably one of a large class. Again, there are plenty of cases in which the appendages are removed when the cause of the suffering was elsewhere. It is needless to say that such cases go to swell the number of failures, but it is the operator who is the failure.

In the treatment of the non-cystic form of oöphoro-salpingitis, the electrician competes with the surgeon, and claims the most brilliant results for electricity. Dr. Sanders, in a recent article, reports twenty-two cures out of twenty-five cases treated. He states that from six months to one year may be required for treatment. In not one of the cases in my list could the patient afford to spend such a length of time in treatment. We hear so much about the eager young operator who wishes to add another victim to his list, but I take it that, apart from a question of conscience, it is distinctly to the physician's advantage to cure his patient, and that none of us can afford to turn loose in the community a number of "mutilated" and complaining women. The electricians never seem to consider that the reason so many salpingo-oöphorectomies are performed is because most patients cannot carry out any long-continued course of treatment. Personally, I had rather cure my patient without an operation, and I would always use various local methods of treatment before resorting to the knife. I have not yet had the good fortune to cure a case of markedly diseased uterine appendages by means of electricity or any other local methods of treatment, where the trouble has existed as great a length of time as in my operative cases. In eleven of my salpingo-oöphorectomies, no other treatment except operation would have been proper. In the other nine, outside of the fatal case, the subsequent history speaks in favor of the treatment pursued. As a rule, the most unsatisfactory results have been in those cases which, month after month, have been subjected to local treatment; not only with no improvement to the diseased structures, but with a distinct impairment of the general health due to long-continued suffering.

It is not my intention to advocate any pet method of treatment, but merely to place these clinical observations on record, and give the general practitioners an opportunity to judge of the question for themselves, by clearing away the smoke of the conflict, and presenting to them the following conclusions regarding operative treatment in cases of diseased appendages:

1. Each case must be judged on its own merits and surrounding circumstances. There is no one form of treatment which will guarantee a cure.

2. Salpingo-oöphorectomy stands on the same level as any other surgical procedure, and its results are influenced by a variety of elements which may play a part after any surgical method of treatment.

3. The results of salpingo-oöphorectomy are sufficiently encouraging to justify its performance after milder methods have failed. The most serious mistakes the physician can make are: (*a*), continuing local treatment after such a course has proven worthless; (*b*), making too positive assertions as to the favorable result of the treatment.

The operation is not a guarantee against poverty or old age, but justice to the patient requires prejudice to be put one side and a fair chance given her to regain her health.

### Abdominal Sections.

| Number. | Date. | History, Symptoms, etc. | Operation. | Pathological Conditions. | Result. | Remarks. |
|---|---|---|---|---|---|---|
| 1 | Aug. 22, '91. | Age, 30; married; 2 children. Gonorrheal infection five years ago; dysmenorrhea and menorrhagia, uterine syndroma. Digestive and nervous system wrecked; firm masses on each side of uterus. | Salpingo-oöphorectomy. | Chronic non-cystic oöphoro-salpingitis. Firm adhesions. | R. | Not seen since operation. |
| 2 | Aug. 29, '91. | Age, 25; married; no pregnancies. Gonorrheal infection; metrorrhagia, high temperature. Large fluctuating masses in pelvis. | Salpingo-oöphorectomy. Irrigation and drainage. | Pus tubes size of large sweet potatoes. | R. | Not seen since operation. |
| 3 | Sept. 23 '91. | Age, 24; married; 2 children; 2 abortions. Chronic endometritis and salpingitis for four years; acute exacerbation following induced abortion. Large inflammatory mass in pelvis. | Salpingo-oöphorectomy. Drainage. | Large hemato-salpinx on right side. Chronic non-cystic inflammation of appendages on left side. | R. | November, 1893. Patient is still troubled with leucorrhea. Her general condition very good. |
| 4 | Jan. 28, '92. | Age, 17; single. Menstruation absent for ten weeks; development of large mass in pelvis. Severe pelvic pains with emaciation. | Salpingo-oöphorectomy. Drainage. | Pyosalpinx on left side, cystic ovary, thick closed tube on right side. Layer of adhesions half an inch thick. | R. | Has since married and is doing hard work. |
| 5 | May 3, '92. | Age, 27; married; 1 child five years ago. Typical history of ectopic gestation, with rupture. (Reported in *Buffalo Medical and Surgical Journal*, July, 1892.) | Removal of right tube and sac. No drainage. | Ruptured right tube, sac and fetus enclosed in fibrinous layer. Fetus at about third month. | R. | Perfect health six months after operation. |

ABDOMINAL SECTIONS.—*Continued.*

| Number. | Date. | History, Symptoms, etc. | Operation. | Pathological Conditions. | Result. | Remarks. |
| --- | --- | --- | --- | --- | --- | --- |
| 6 | May 5, '92. | Age, 30; married. Chronic metritis and salpingitis for two years; last two months confined to bed. Examination showed chronic inflammation of appendages. | Salpingo-oöphorectomy. | Extensive adhesions, chronic non-cystic ovaritis and salpingitis. | R. | Lost sight of. |
| 7 | May, 1892. | Age, 25; married; 2 children; 1 abortion. Pelvic peritonitis following abortion, pain, fever; confined to bed past six weeks. Large tender mass to right of uterus. | Exploratory, freeing intestinal adhesions. Perforated, friable cecum fastened in a secondary abdominal incision. Drainage. | Appendages normal; cecum and mass of intestines adherent to right broad ligament. Several holes torn in cecum. No pus. | R. | Patient is going out as washerwoman. Has ventral hernia. Is otherwise well. |
| 8 | May 25, '92. | Age, 25; 1 child seven years ago. Leucorrhea, backache, abdominal pain, and menorrhagia since birth of child; local treatment for years. Small cyst on left side of uterus, indurated mass on right side. | Salpingo-oöphorectomy. | Very dense adhesions, chronic non-cystic oöphoro-salpingitis. Cyst size of hen's egg in left broad ligament. | R | Required treatment for leucorrhea. November 1, 1893, reports herself as entirely well and able to do hard work. |
| 9 | June 23, '92. | Age, 37; single. Tumor noticed during past year; pain and emaciation. Solid tumor, extending half way to umbilicus. | Exploratory. | Malignant tumor springing from pelvis. Unusually adherent. | R. | Nearly died from the anesthetic; was failing rapidly when discharged. |
| 10 | July 5, '92. | Age, 36; married; 6 children; 1 abortion. For past six months | Salpingo-oöphorectomy. Drainage. | Double ovarian abscess, pelvic peritonitis. | R. | One year after operation is doing all her own |

| | | | | | | |
|---|---|---|---|---|---|---|
| | | just able to drag herself about; abdominal pains, backache, etc.; acute exacerbation following abortion. Pelvis filled with inflammatory mass. | | | | housework; is not strong but has no pelvic symptoms. |
| 11 | July 9, '92. | Age, 25; married; 1 child two and one-half years ago. Adherent retroverted uterus; chronic endometritis and salpingitis since birth of child. Confined to bed most of the past year. | Salpingo-oöphorectomy. Hysterorrhaphy. | Dense adhesions—retroversion, chronic, non-cystic oöphoro-salpingitis. | D. | Died on third day from septic peritonitis; no cause discoverable. |
| 12 | July 9, '92. | Age, 32; married. Gonorrhea contracted from husband several years ago; acute exacerbation. Pelvis filled with a large mass. | Salpingo-oöphorectomy. Irrigation and drainage. | Double pyosalpinx. | R. | Patient reported herself as quite well three months after operation. |
| 13 | July 26, '92. | Age, 23; married; 1 child ten weeks ago. Septic infection following labor; several weeks of fever with numerous chills. Large mass to right of uterus. Temperature 102°, pulse 138, at time of operation. | Exploratory. Drainage. | Pelvic peritonitis, extensive adhesions. Mass formed by distended right broad ligament. Appendages normal. | D. | Death third day from continuance of disease. Post-mortem showed multiple suppurating foci in sub-peritoneal tissue of abdominal wall; no pus in cellular tissue of pelvis. Route of infection followed round ligament to sub-peritoneal tissue. |
| 14 | Dec. 20, '92. | Age, 24; single. Past six months abdominal pains, menorrhagia, dysmenorrhea; is unable to work. Possible gonorrheal history. | Salpingo-oöphorectomy. Drainage. | Extensive adhesions, chronic, non-cystic oöphoro-salpingitis. | R. | Has been doing hard work. Is perfectly well except for small hernia at site of drainage-tube opening. |

### Abdominal Sections.—*Continued.*

| Number. | Date. | History, Symptoms, etc. | Operation. | Pathological Conditions. | Result. | Remarks. |
|---|---|---|---|---|---|---|
| 15 | Mar. 16, '93. | Age, 36; married; 3 children; 1 abortion. Ill since abortion six months ago; pains in abdomen, fever, emaciation; confined to bed. Large mass on right side of pelvis. | Drainage of pelvic abscess. | Everything matted together. Deep down among intestines in front of right broad ligament collection of pus. | R. | Very much improved, able to take care of house. At times considerable abdominal pain (Adhesions?) |
| 16 | Mar. 21, '93. | Age, 20; prostitute. Frequent attacks of pelvic peritonitis. Large tender masses in pelvis. | Salpingo-oöphorectomy. Drainage. | Dense adhesions. Ovarian abscess on one side. Large ovarian hematoma on other side. Tubes closed and thickened. | R. | Good health considering she continues her old life. Entire loss of sexual desire and enjoyment. |
| 17 | Mar. 23, '93. | Age, 28; married; 4 children. For years backache, leucorrhea, etc.; since birth of last child, six months ago, considerable pelvic pain, incapacitated for work. Mass size of a man's fist lying to left of uterus. | Salpingo-oöphorectomy. Left side. Drainage. | Pyosalpinx, left side. Right side apparently normal. | R. | November 1, 1893. Is doing her entire housework for first time in six years. Is pregnant two months. |
| 18 | May 18, '93. | Age, 44; married; 2 children. Never well since birth of first child ten years ago; severe backache, pain in left side. Uterus retroverted and fixed, ovaries prolapsed. | Salpingo-oöphorectomy. Hysterorrhaphy. | Adhesions numerous and dense. Chronic, non-cystic oöphoro-salpingitis. | R. | Three months after operation was better than at any time during past ten years. Is improving in general health. |
| 19 | Aug. 1, '93. | Age, 21; married; 2 abortions. Trouble caused by inducing the | Salpingo-cöphorectomy. | Numerous adhesions. Chronic, non-cystic oöpho- | R. | November 1, 1893. Is able to support herself as |

| | | | | | | |
|---|---|---|---|---|---|---|
| | | abortions; for past six weeks severe pains in abdomen; unable to be upon her feet. Tender mass on each side of uterus. | | ro-salpingitis. | | cook in a restaurant. |
| 20 | Aug. 2, '93. | Age, 45; married; 6 children. Ordinary history of neglected cancer of uterus. Cancerous mass filling half the vagina. | Amputation of cancerous mass. Supra-vaginal hysterectomy. Drainage. | Cancer involving uterus and top of vagina. | R. | Acute mania following operation. Recovery. |
| 21 | Aug. 8, '93. | Age, 38; married; 3 children; 4 abortions. Past two months severe backache and pain in left side. A large doughy mass filling Douglas' pouch. | Salpingo-oöphorectomy. | Multiple follicular cysts of both ovaries forming masses size of a man's fist. | R. | Lost sight of. |
| 22 | Aug. 15, '93. | Age, 32; married; 2 children. For nine weeks severe pelvic pains; metrorrhagia; not able to be about. Pelvis filled with mass size of child's head. | Curetting of uterus. Salpingo-oöphorectomy. Suture of peritoneal coat of gut. Drainage. | Universal adhesions. Large hemato-salpinx on right side. Pyosalpinx on left side. | R. | Is able to support herself by going out washing. |
| 23 | Aug. 15, 93. | Age, 32; married; 3 children; 1 abortion. Several years of ill-health; for past month pelvic inflammation, with inability for work. Large inflammatory masses in pelvis. | Curetting of uterus. Salpingo-oöphorectomy. Drainage. | Pyosalpinx and ovarian abscess on both sides. | R. | General health not yet good; greatly improved and able to work. |
| 24 | Aug. 22, '93. | Age, 19; prostitute. Abortion six months ago; since then severe pains in abdomen, requiring large amount of morphine. Uterus retroverted, and imbedded in an inflammatory mass. | Curetting of uterus. Salpingo-oöphorectomy. Hysterorrhaphy. Drainage. | Double pyosalpinx with numerous adhesions. | R. | November 1, 1893. Reports herself as quite well and reformed. |

### Abdominal Sections.—*Continued.*

| Number. | Date. | History, Symptoms, etc. | Operation. | Pathological Conditions. | Result. | Remarks. |
|---|---|---|---|---|---|---|
| 25 | Aug. 22, '93. | Small ventral hernia in site of drainage-tube opening in case No. 14. | Adhesions of omentum and gut freed. Fascia closed by silkworm gut. | | R. | |
| 26 | Aug. 29, '93. | Age, 21; prostitute. Three attacks of pelvic peritonitis during the past year. Inflammatory mass in pelvis. | Curetting of uterus. Salpingo-oöphorectomy. | Dense adhesions. Chronic non-cystic oöphoro-salpingitis. | R. | November 1, 1893. States that she is perfectly well in every respect. |
| 27 | Sept. 2, '93. | Age, 34; 2 children. History of gradual growth of a pelvic tumor. Cystic tumor size of a man's head. | Ovariotomy. Drainage. | Intraligamentous cyst of right ovary; weight 9 lbs. Pelvic peritoneum up and mesentery opened by tumor. | R. | |
| 28 | Sept. 24, '93. | Age, 35; single. Ordinary history of cancer of the uterus. About one-half the cervix affected. | Supra-vaginal hysterectomy. | Carcinoma of cervix. | R. | |
| 29 | Oct. 14, '93. | Age, 41; 3 children. History of rupture several weeks ago; abdominal pain; irregular flowing since. Pelvis filled with tumor. | Salpingo-oöphorectomy. Ovariotomy. | Ruptured tubal pregnancy right side; cyst size of large orange on left side. | R. | |
| 30 | Oct. 18, '93. | Age, 39; 3 children. Seven years of backache, pain in left side, leucorrhea, menorrhagia. Retroverted adherent uterus, prolapsed ovaries. | Curetting. Trachelorrhaphy perineorrhaphy Salpingo-oöphorectomy. Hysterorrhaphy. | Numerous adhesions, cystic oöphoritis, chronic salpingitis. | R. | |

